# Protein kinase D1 regulates innate immune signaling pathways to promote hepatitis C virus replication

**DOI:** 10.1128/jvi.00693-26

**Published:** 2026-09-09

**Authors:** Jiyoung Lee, Yu-Chen Chuang, Johanna Ahn Lee, J.-H. James Ou

**Affiliations:** 1 Department of Immunology and Immune Therapeutics, University of Southern California, Keck School of Medicine 12223, Los Angeles, California, USA; Wake Forest University School of Medicine, Winston-Salem, North Carolina, USA

**Keywords:** HCV replication, inflammasome, TNF-alpha, interferon response, protein kinase D1, hepatitis C virus

## Abstract

Hepatitis C virus (HCV) has developed multiple means to disrupt host innate immune response to promote its replication. Protein kinase D1 (PKD1) is a serine/threonine kinase involved in cell proliferation and lipid metabolism. By analyzing the effect of PKD1 on HCV replication, we found that HCV activated PKD1 via protein kinase C (PKC) early in infection and induced the cleavage of PKD1 at later timepoints of infection via caspase-3. PKD1 silencing had no effect on HCV entry, but it reduced HCV RNA levels in cells, indicating an important role of PKD1 in promoting HCV replication. PKD1 silencing also induced the expression of interferons (IFNs) and IFN-stimulated genes (ISGs) and augmented the HCV-induced TNF-α expression, which enhanced the IFN response. Interestingly, the induction of inflammasomes was compromised in HCV-infected cells in the absence of PKD1, indicating an important role of PKD1 in promoting the inflammasomal response. Taken together, our results indicated that HCV activated PKD1 to suppress the IFN response and TNF-α signaling and in the meantime induce the inflammasomal response. Our results, thus, revealed an interesting interplay between HCV and host innate immune responses and an important role of PKD1 in this intricate interplay.

**IMPORTANCE:**

Hepatitis C virus (HCV) establishes chronic infection in most patients that it infects, and this chronic infection can lead to severe liver diseases including steatosis, cirrhosis, and hepatocellular carcinoma. PKD1 is a multifunctional protein that regulates lipid metabolism, cell proliferation, and cellular apoptosis. In this report, we investigated the relationship between HCV and PKD1. We found that HCV could activate PKD1 to suppress interferon response and TNF-α signaling to promote its replication and in the meantime enhance the inflammasomal response. These effects of HCV on PKD1 provide important insights for understanding the mechanism of HCV persistence and pathogenesis in patients.

## INTRODUCTION

Hepatitis C virus (HCV) is an important human pathogen. There are approximately 58 million people worldwide that are chronically infected by this virus, with ~1.0 million new cases every year (1). The infection by HCV frequently leads to a lifelong chronic infection, which can result in the development of severe liver diseases including steatosis, cirrhosis, and hepatocellular carcinoma. Although efficacious treatments are available for HCV patients, many of them are not aware of the infection until severe disease symptoms have developed (1). HCV is a positive-stranded RNA virus with a genome size of 9.7 kb. Its genome contains a long open reading frame that codes for a polyprotein that is slightly more than 3,000 amino acids in length. This polyprotein is cleaved by viral and cellular proteases to generate 10 mature protein products, with the structural proteins located at the N-terminus and nonstructural (NS) proteins located at the C-terminus (2).

How HCV evades host immunity to establish chronic infection is an interesting question. Pattern-recognition receptors (PRRs) are sensors of non-self-molecular signatures called pathogen-associated molecular patterns (PAMPs) and molecular triggers of antiviral responses. The three classes of PRRs are the retinoic acid-inducible gene-I (RIG-I)-like receptors (RLRs), Toll-like receptors (TLRs), and nucleotide-binding oligomerization domain (NOD)-like receptors (NLRs). HCV PAMPs for RIG-I are the 5′-PPP double-stranded RNA and the 3′ poly U/UC sequence in the genomic RNA (3). After the activation by HCV PAMPS, RIG-I interacts with the mitochondrial adaptor protein MAVS on the mitochondrial membrane. Once activated, MAVS then recruits TRAF3 and TRAF6. This leads to the activation of the downstream TANK-binding kinase 1 (TBK1), which will phosphorylate and activate Interferon Regulatory Factor 3 (IRF3). The IRF3 homodimer can then be translocated to the nucleus to transactivate the expression of interferons (IFNs) (4). The interplay between HCV and host innate immune responses at the molecular level has been of great interest, and HCV has been shown to use multiple means to disrupt the signaling pathways of innate immune responses. Autophagy is a catabolic process that is important for removing protein aggregates and damaged organelles for maintaining cellular homeostasis. It can also remove intracellular microbial pathogens via a process known as xenophagy (5). HCV has also been shown to subvert this cellular pathway and use it to promote its RNA replication and the degradation of TRAF6 (6–8). HCV-induced autophagy also removes tumor necrosis factor receptor 1 (TNFR1), which is required for TNF-α signaling to sensitize cells to type 1 IFNs (9), and type 1 IFN receptor IFNAR1 to inhibit the IFN response (9, 10).

PKD1 is a serine/threonine kinase that is encoded by the PRKD1 gene. PKD1, previously called protein kinase C µ (mu), contains an N-terminal regulatory domain and a C-terminal catalytic domain. The binding of diacylglycerol (DAG) to PKD1 facilitates its translocation to the membrane for its subsequent phosphorylation at serine 738/742 and activation by PKC (11, 12). PKD1 regulates the secretory process, lipid metabolism, and proliferation of the cell (13–16). PKD1 can be cleaved by caspase-3, the executioner caspase of apoptosis, at Asp-378 for the release of the 60-kDa C-terminal catalytic fragment and sensitizes cells to apoptosis induced by genotoxic stress (17). During HCV infection, PKD1 phosphorylates ceramide transfer protein (CERT) and oxysterol-binding protein (OSBP) to suppress their functions, thereby reducing the vesicular trafficking to the plasma membrane (18). This also reduced the secretion of HCV, likely due to the negative effect of PKD1 on the secretion pathway from Golgi to the plasma membrane (18).

In this report, we further studied the relationship between HCV and PKD1. To our surprise, we found that HCV could activate PKD1 to support its replication. The effect of PKD1 on HCV replication was via the inhibition of IFN and TNF-α signaling pathways. Interestingly, PKD1 also enhanced the inflammatory response in HCV-infected cells.

## RESULTS

### HCV induces the phosphorylation of PKD1

**Fig 1:**
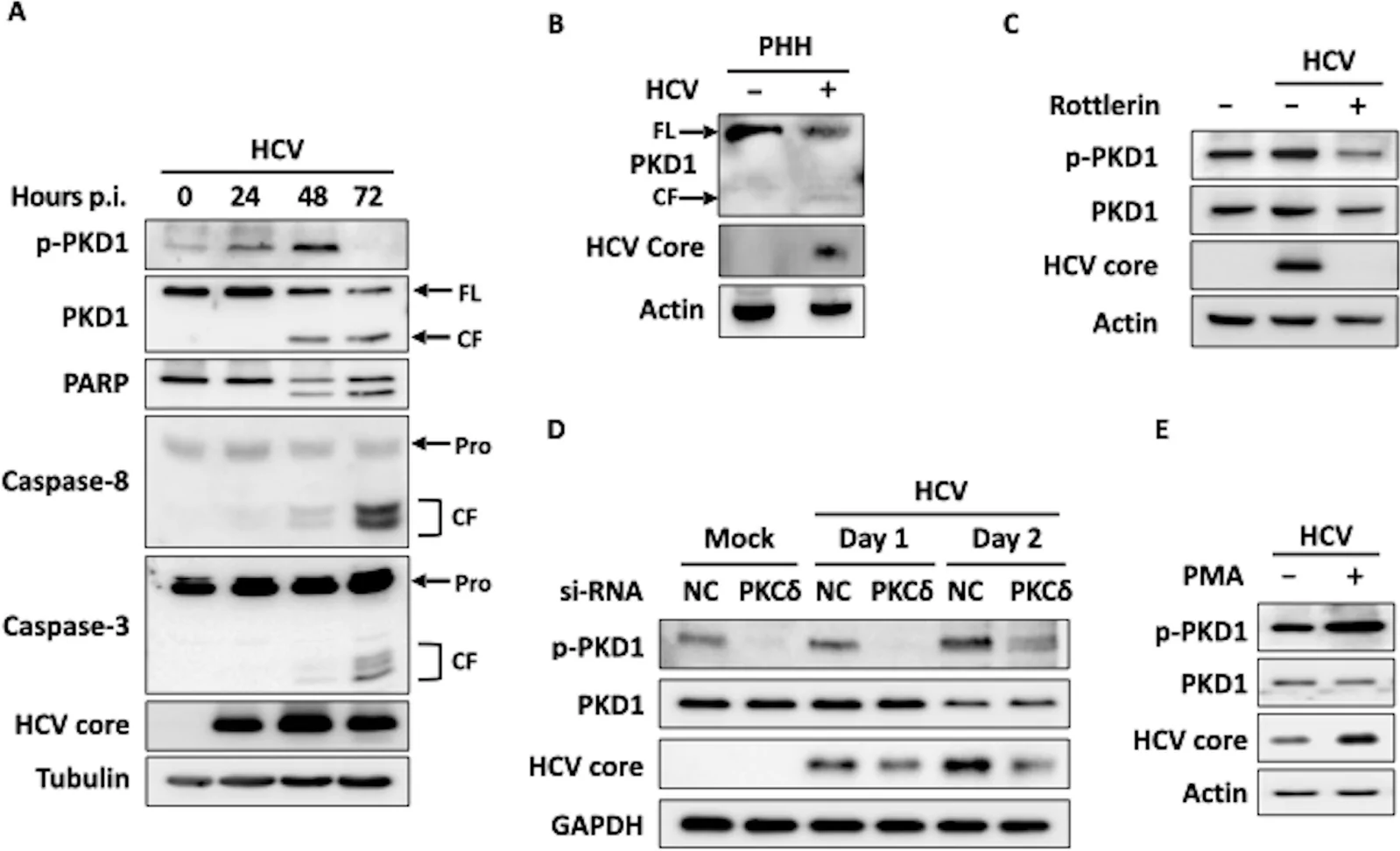
HCV induces the phosphorylation and cleavage of PKD1. (**A**) Huh7 cells were infected with HCV (MOI = 0.25). Total cell lysates were collected at the indicated timepoints and subjected to SDS-PAGE and immunoblotting. FL, full-length PKD1; CF, cleaved fragment; Pro, procaspase-3 and 8. (**B**) Primary human hepatocytes were mock- or HCV-infected (MOI = 0.25) and lysed at 24 h post-infection for immunoblot analysis. The result shown was part of a previously published study (9) and was reused for analysis of PKD1 cleavage. (**C**) Huh7 cells were pretreated with DMSO or 5 μM Rottlerin for 1 h before infection with HCV (MOI = 0.25). Cells were lysed at 24 h post-infection for immunoblot analysis. (**D**) Huh7 cells were transfected with either negative control siRNA (NC) or PKCδ siRNA for 48 h, followed by a second transfection with the same siRNA for an additional 6 h. The cells were then infected with HCV and harvested at either 24 h or 48 h post-infection, as indicated. (**E**) Huh7 cells were infected with HCV (MOI = 1). The following day, cells were treated with DMSO or PMA (100 nM) overnight before immunoblot analysis.

To examine the relationship between HCV and PKD1, we infected Huh7 cells, a human hepatoma cell line, with a cell culture-adapted variant of the HCV JFH1 strain (19). As shown in Fig. 1A, HCV induced the phosphorylation of PKD1 at Ser738/742, which was visible at 24 h and peaked at 48 h post-infection. The phosphorylated PKD1 was no longer detectable at 72 h post-infection although this loss of phosphorylation was associated with the cleavage of PKD1, which was also detectable at 48 h post-infection. The cleavage of PKD1 coincided with the induction of apoptosis, as evidenced by the cleavage of poly(ADP-ribose) polymerase (PARP), caspase-8, and caspase-3 (Fig. 1A), which are markers of apoptosis. As the HCV core protein level also peaked at 48 h and reduced at 72 h, there was a positive correlation between the phosphorylation of PKD1 and the level of the HCV core protein. HCV also induced the cleavage of PKD1 in primary human hepatocytes (PHHs) at 24 h post-infection (Fig. 1B).

Since PKC phosphorylates PKD1 at Ser738 and Ser742, we tested the possible role of PKC in the phosphorylation of PKD1. As shown in Fig. 1C, the treatment of HCV-infected cells with Rottlerin, a well-established PKCδ inhibitor (20), reduced the phosphorylation of PKD1. It also reduced the HCV core protein to an undetectable level. To rule out the possible nonspecific effect of Rottlerin, we also silenced the expression of PKCδ with siRNA (Fig. S1). As shown in Fig. 1D, this silencing markedly reduced the phosphorylation of PKD1 in both mock-infected and HCV-infected cells. It also reduced the HCV core protein level in HCV-infected cells. In contrast, the treatment of HCV-infected cells with phorbol myristate acetate (PMA), a well-established activator of PKC (21), increased PKD1 phosphorylation and the core protein level (Fig. 1E). These results supported the role of PKC in the phosphorylation and activation of PKD1 in HCV-infected cells and further indicated a positive correlation between PKD1 activation and HCV replication.

### HCV-induced cleavage of PKD1 is mediated by caspase-3

**Fig 2:**
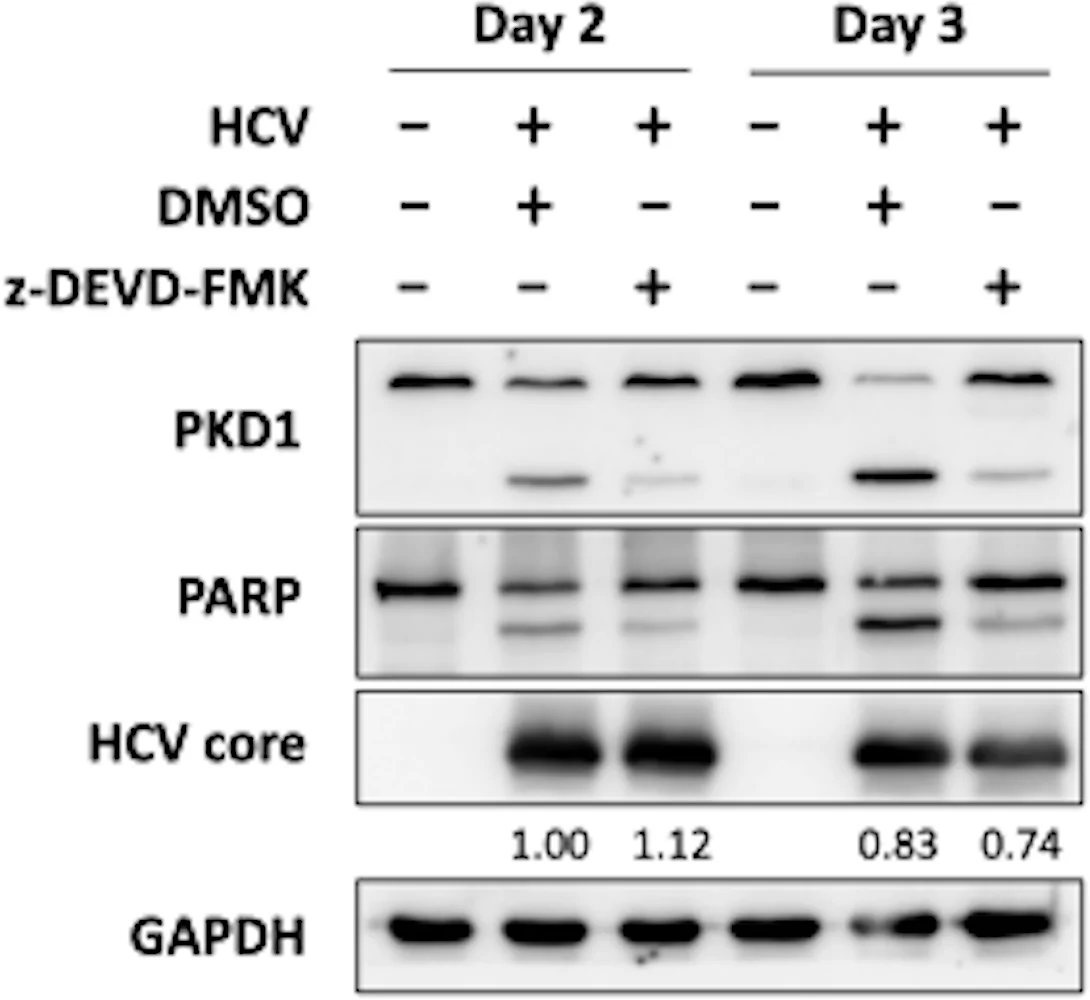
Inhibition of HCV-induced cleavage of PKD1 by caspase-3 inhibitor. Huh7 cells with and without HCV infection for 2 or 3 days were treated with DMSO or the caspase-3 specific inhibitor z-DEVD-FMK for 16 h and then lysed for immunoblot analysis. The relative HCV core protein levels after normalization against the loading control GAPDH were shown. The HCV core protein level on day 2 with the treatment of DMSO was arbitrarily defined as 1.

It had previously been shown that caspase-3 could mediate the cleavage of PKD in cardiomyocytes under low glucose conditions (22). To test whether the cleavage of PKD1 during HCV infection was also due to the activity of caspase-3, which was also activated in the later stage of HCV infection (Fig. 1A), we treated HCV-infected cells with z-DEVD-FMK, a caspase-3 inhibitor. As a positive control, we also analyzed PARP, a substrate of caspase-3. As shown in Fig. 2, the cleavage of PARP was inhibited by z-DEVD-FMK, confirming the inhibitory effect of this drug against caspase-3. Z-DEVD-FMK also suppressed the cleavage of PKD1, supporting the role of caspase-3 in the cleavage of PKD1 in HCV-infected cells.

Taken together, our results shown in Fig. 1 and 2 demonstrated that HCV induced the phosphorylation and activation of PKD1 during the early phase of infection and the cleavage of PKD1 via caspase-3 in the later phase of infection.

### PKD1 enhances HCV infection

**Fig 3:**
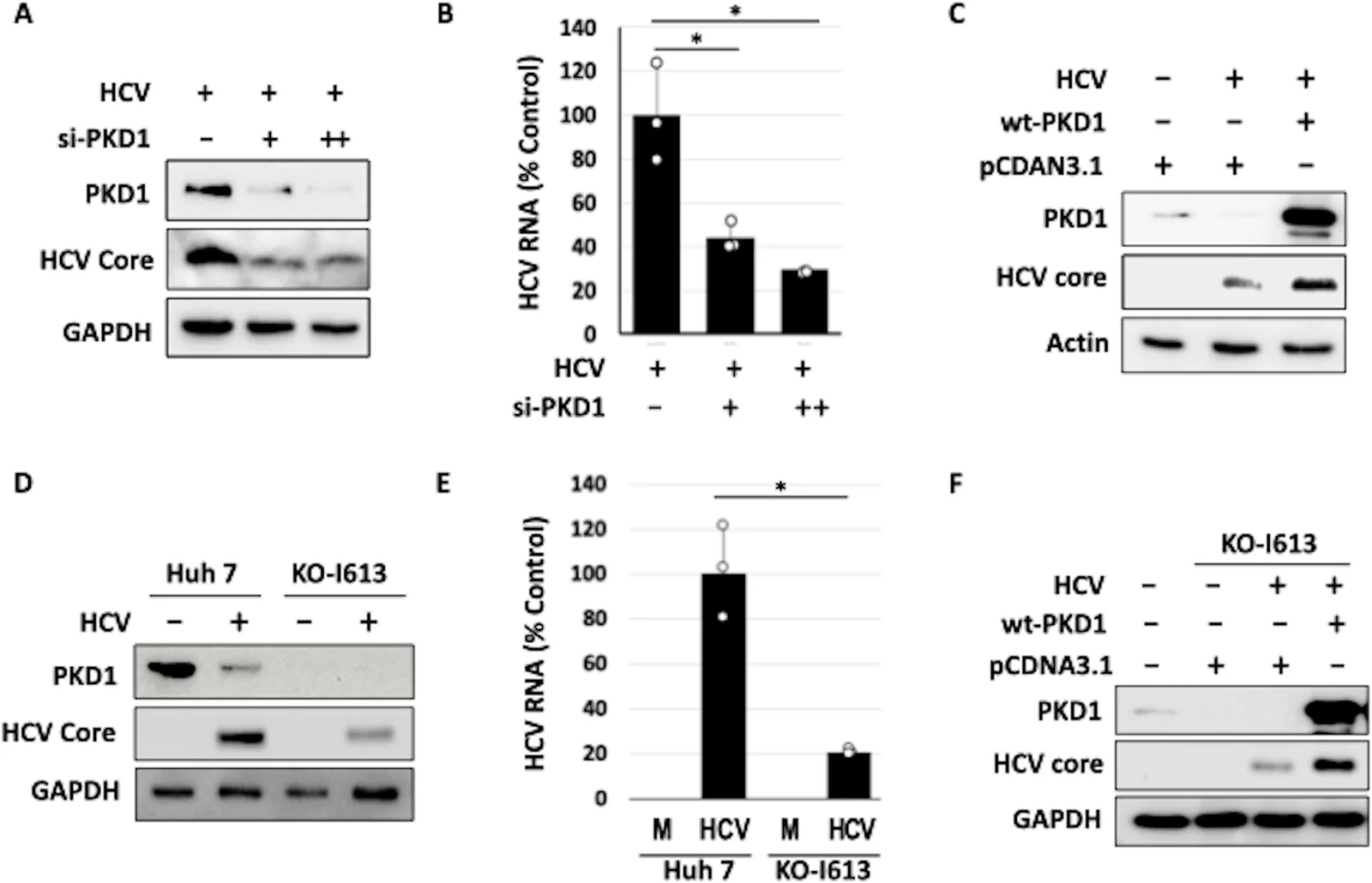
PKD1 enhances HCV infection. (**A and B**) Huh7 cells were transfected with the control siRNA or PKD siRNA for 48 h and then transfected again with the siRNA. Six hours after the second transfection, cells were infected with HCV (MOI = 1). (**A**) Cells were lysed at 24 h post-infection for immunoblot analysis. (**B**) Total cellular RNA was isolated for qRT-PCR using primers specifically targeting JFH1 or GAPDH. The HCV RNA copy number was normalized against that of GAPDH RNA. The data represent an average of three independent experiments. (**C**) Expression of exogenous PKD1. Huh7 cells were transfected with the control vector or the plasmid that expressed HA-tagged human PKD1 (Addgene, Cambridge, MA). The following day, the cells were infected with HCV for 24 h before they were lysed for immunoblot analysis. (**D and E**) Huh7 and KO-I613 cells were infected with HCV (MOI = 1) for 24 h before lysis to obtain total cell lysates for immunoblotting (**D**) and total RNA for qRT-PCR (**E**). (**F**) KO-I613 cells were transfected with the control vector or the plasmid that expressed PKD1. The following day, the cells were infected with HCV for 24 h before they were lysed for immunoblot analysis. *, *P* < 0.05.

To further investigate the role of PKD1 in HCV replication, we first silenced the expression of PKD1 in Huh7 cells with siRNA. The silencing of PKD1 decreased the expression level of the HCV core protein (Fig. 3A) and the intracellular level of HCV genomic RNA (Fig. 3B). In contrast, the over-expression of exogenous PKD1 in Huh7 cells enhanced the expression of HCV core protein (Fig. 3C). To confirm the role of PKD1 in HCV replication, we generated a Huh7 cell line with PKD1 knockout (KO-I613) using CRISPR/Cas9 (for details, see the Materials and Methods section and Fig. S2). The KO-I613 cells were then infected with HCV. Consistent with the result of the siRNA knockdown, the PKD1 knockout decreased the expression level of the HCV core protein (Fig. 3D) as well as the intracellular level of HCV genomic RNA (Fig. 3E). The effect of PKD1 knockout on HCV was not due to the off-target effects of CRISPR/Cas9, as the reintroduction of PKD1 into KO-I613 cells using an expression plasmid increased the HCV core protein level again (Fig. 3F). These results together demonstrated that PKD1 played a positive role in promoting HCV replication.

### PKD1 enhances the replication of HCV without affecting the entry

**Fig 4:**
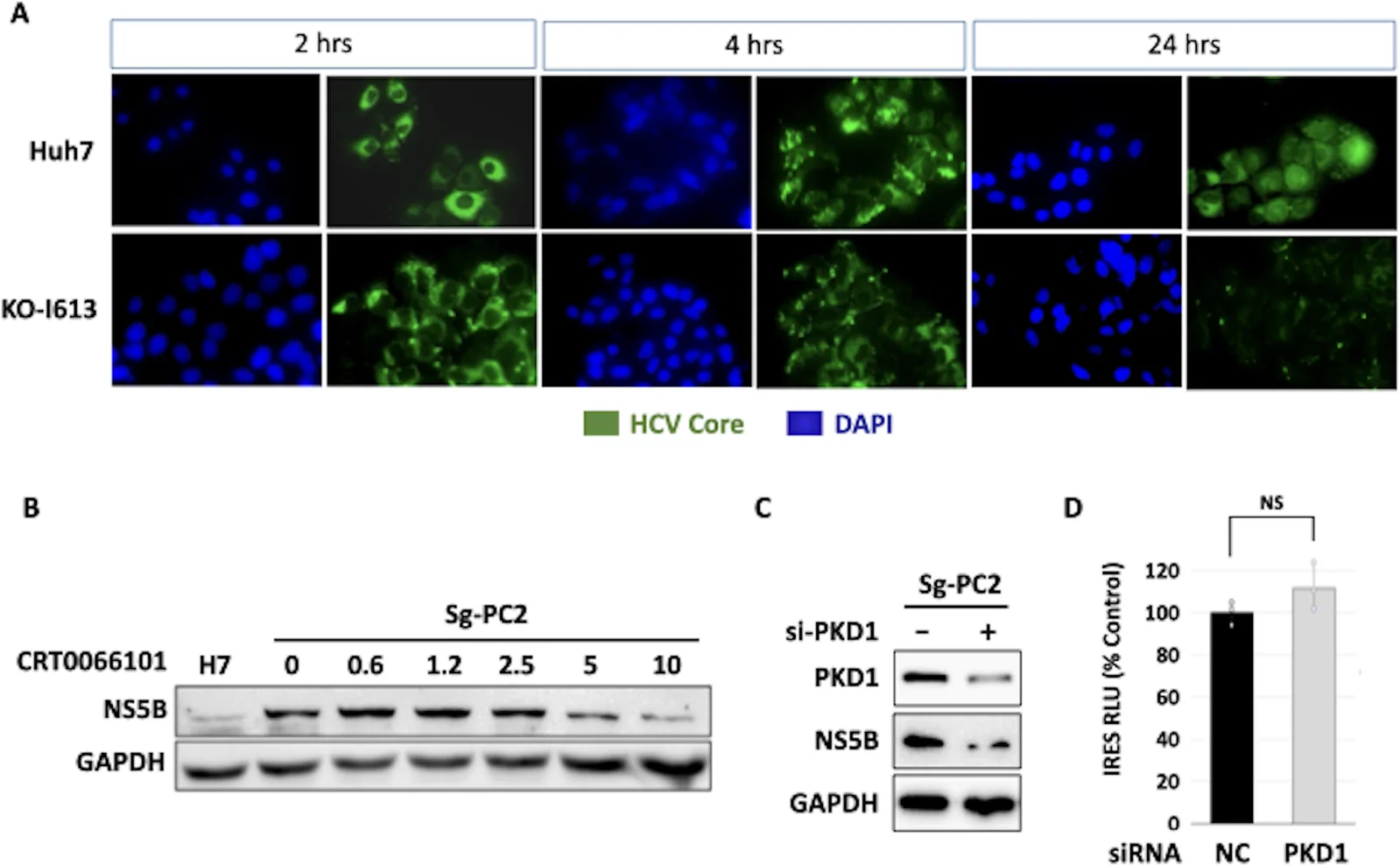
PKD1 enhances HCV replication but not viral entry. (**A**) Huh7 and KO-I613 cells were infected with HCV (MOI = 1) for 2 h. Cells were then rinsed three times with PBS to remove the inoculum before they were fixed for immunostaining. For cells that were infected for 4 or 24 h, the HCV inoculum was removed after 3 h of infection. Cells were then rinsed three times with PBS and further incubated in growth media before fixation for immunostaining. (**B**) Sg-PC2 cells were treated with different concentrations of CRT0066101 for 5 h before lysis. Total lysates from parental Huh7 cells were used as a negative control. (**C**) Sgpc-2 cells were transiently transfected with control siRNA or siRNA targeting PKD1. Cells were lysed 48 h post-transfection for immunoblot analysis. (**D**) Huh7 cells transfected with the control siRNA or the PKD1 siRNA were transfected with the bicistronic luciferase reporter pRL-HL. The relative HCV IRES activities were measured based on the firefly luciferase activities after normalization against the renilla luciferase control. NS indicates *P* > 0.05.

To further investigate how PKD1 affected HCV replication, we conducted the immunofluorescent staining experiment of the HCV core protein in Huh7 cells and KO-I613 cells that were infected by HCV. As shown in Fig. 4A, there was no discernible difference of the HCV core protein signals between these two cell lines at 2 and 4 h post-infection. However, the HCV core protein signal was greatly diminished in KO-I613 cells at 24 h post-infection. These results indicated that PKD1 likely did not affect the entry of HCV into its host cells, and its effect on HCV was most likely at a post-entry step.

Sg-PC2 is a stable Huh7 cell line that contains an HCV subgenomic RNA replicon that does not express HCV structural proteins (23). To determine how PKD1 enhanced HCV replication after viral entry, we treated Sg-PC2 cells with different doses of CRT0066101, a PKD1-specific inhibitor. As shown in Fig. 4B, the expression level of the HCV NS5B protein was reduced in Sg-PC2 cells by CRT0066101 when the drug concentration used reached 5 μM. This reduction was further enhanced when the drug concentration was increased to 10 μM. As CRT0066101 might have other nonspecific effects, we also silenced the expression of PKD1 in Sg-PC2 cells with siRNA. This silencing also significantly reduced the HCV NS5B protein level in Sg-PC2 cells (Fig. 4C). To determine whether PKD1 affected the translation of HCV RNA, which is mediated by the HCV internal ribosomal entry site (IRES), we transfected Huh7 cells with the DNA plasmid pRL-HL, which expressed a bicistronic RNA that contained the renilla luciferase coding sequence at the 5′-end and the firefly luciferase coding sequence at the 3′-end. In this bicistronic RNA construct, the translation of the renilla luciferase was cap-dependent whereas that of the firefly luciferase was mediated by the HCV IRES. The former served as the internal control to monitor the transfection efficiency. As shown in Fig. 4D, the silencing of PKD1 with siRNA had no effect on the HCV IRES activity in Huh7 cells. These results indicated that PKD1 played an important role in promoting HCV RNA replication.

### PKD1 enhances HCV infection via inhibition of the TBK1/IRF-3/IFN pathway

**Fig 5:**
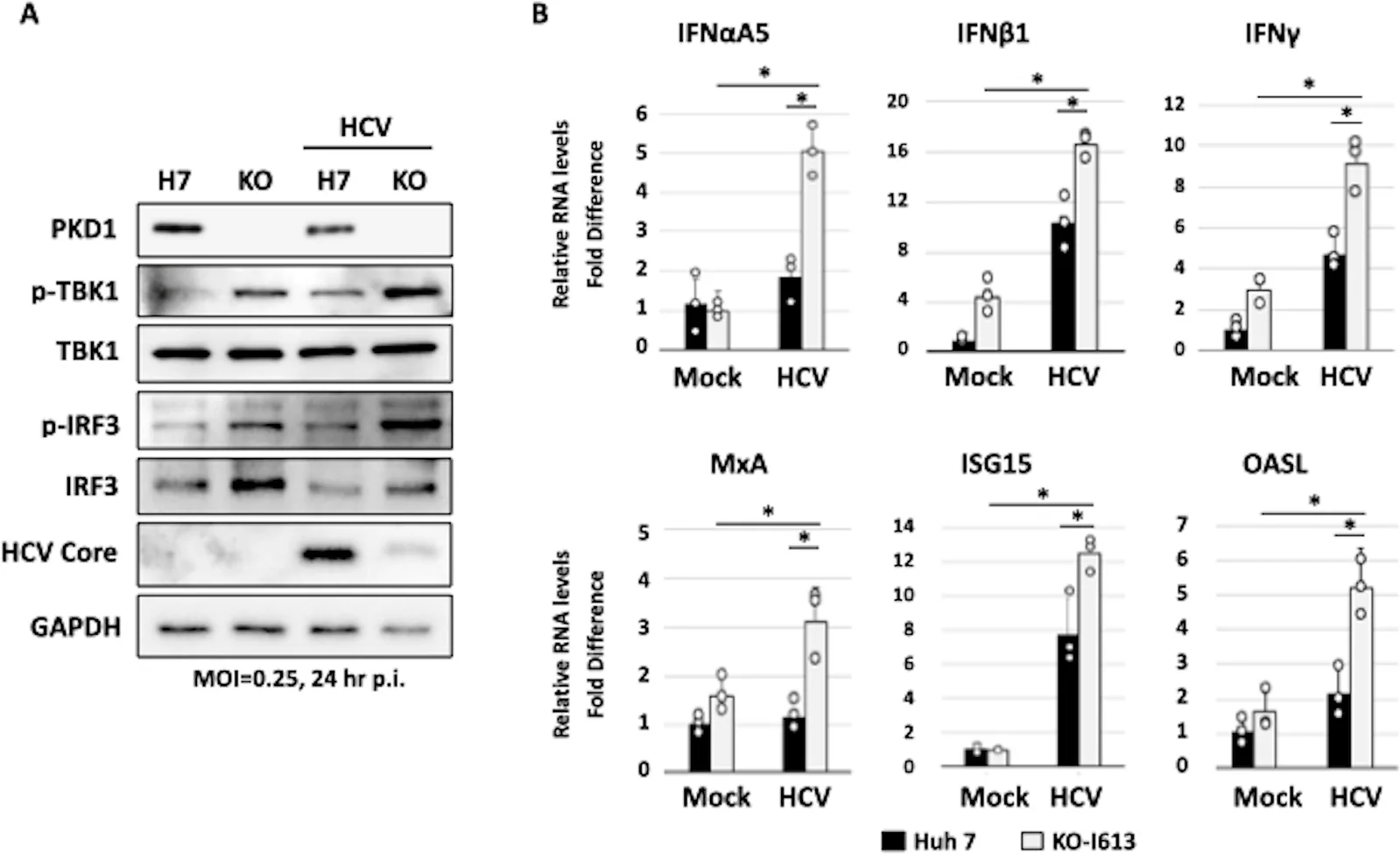
PKD1 promotes HCV infection via inhibition of the IFN pathway. Huh7 or KO-I613 cells were infected with HCV (MOI = 0.25). After 24 h of infection, cells were lysed to obtain total cell lysates and for the isolation of total RNA. (**A**) Immunoblot analysis of cellular proteins and the HCV core protein. (**B**) One hundred nanograms of total RNA was used for qRT-PCR analysis of IFN and ISG RNAs. * indicates *P* < 0.05.

HCV is known to disrupt host innate immune responses to stimulate its own replication. For that reason, we tested the possible relationship between PKD1 and intracellular antiviral responses. The activation of PRRs can trigger the downstream signaling to activate TBK1, which will then phosphorylate and activate IRF3 (24). As shown in Fig. 5A, PKD1 knockout increased the phosphorylation of TBK1 and IRF3 as well as the level of total IRF3, indicating a negative role of PKD1 in the regulation of the TBK1-IRF3 pathway. HCV slightly increased the phosphorylation of TBK1 and IRF3 in Huh7 cells. Interestingly, this HCV-induced phosphorylation of TBK1 and IRF3 was enhanced in KO-I613 cells. The activation of TBK1 and IRF3 in the KO-I613 cells was concomitant with a decrease of the HCV core protein level, in agreement with the result shown in Fig. 3D. These results indicated that PKD1 suppressed the TBK1-IRF3 signaling pathway in both mock-infected and HCV-infected cells.

The activation of IRF3 could lead to the induction of IFNs. We, therefore, compared the mRNA levels of IFNs and ISGs in both Huh7 and KO-I613 cells with and without HCV infection. The knockout of PKD1 in KO-I613 cells had no effect on IFN-αA5 expression in mock-infected cells. However, it increased the expression of IFN-β1 and IFN-γ (Fig. 5B). In contrast, HCV induced the expression of IFN-αA5, IFN-β1, and IFN-γ in both Huh7 and KO-I613 cells with a greater effect in the latter. Accordingly, the expression levels of ISGs including MxA, ISG15, and OASL were also higher in HCV-infected cells, with greater effects in KO-I613 cells (Fig. 5B). Collectively, the results shown in Fig. 5 demonstrated that PKD1 suppressed the TBK1/IRF3/IFN signaling pathway, and its depletion potentiated the HCV-induced IFN response, resulting in the suppression of HCV replication.

### PKD1 restricts the IFN response by inhibiting the production of TNF-α

**Fig 6:**
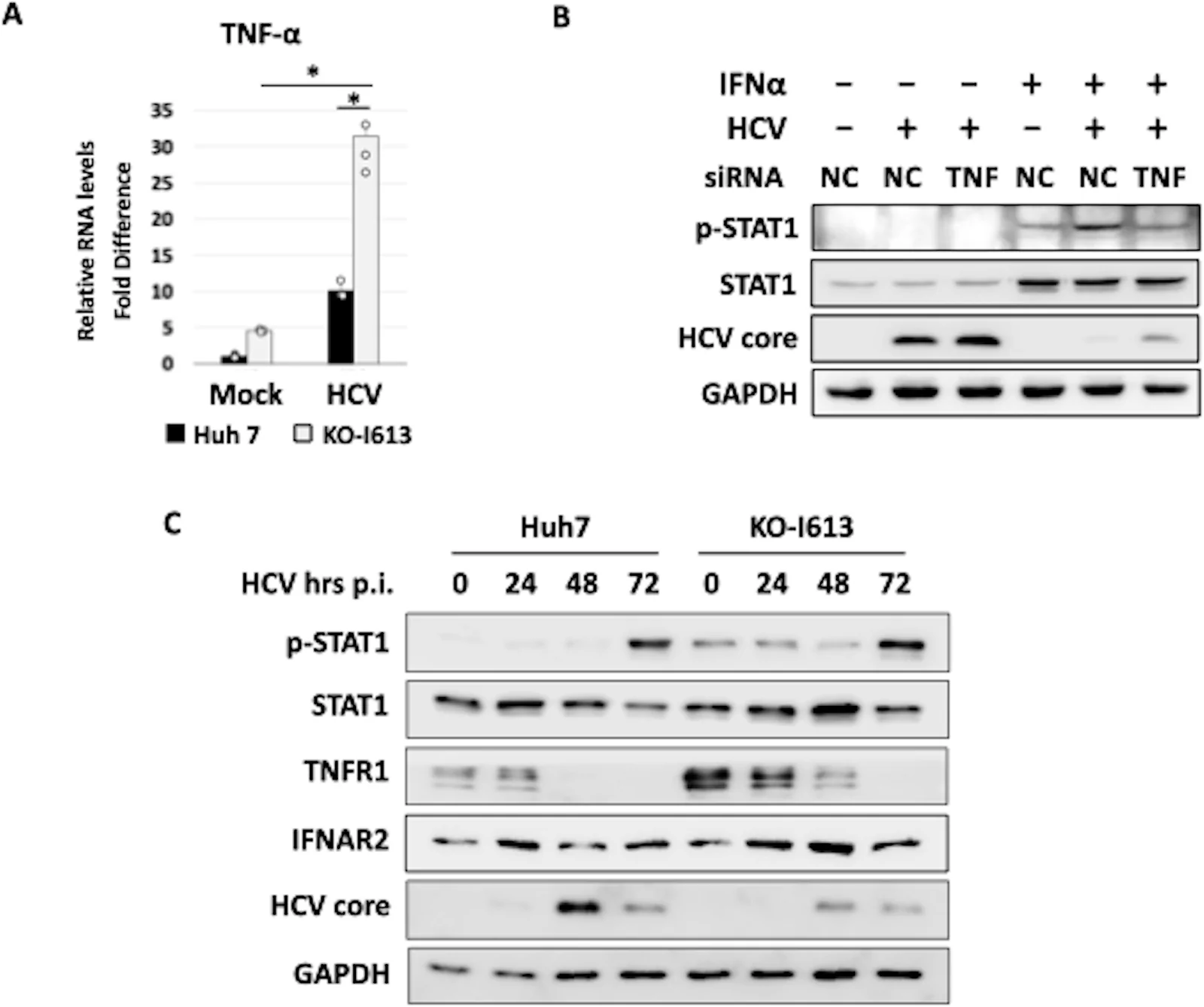
PKD1 attenuates the production of TNF-α to restrict IFN response. (**Α**) Huh7 and KO-I613 cells were mock-infected or infected with HCV for 24 h. Total RNA was then isolated for qRT-PCR analysis of TNF-α RNA. * indicates *P* < 0.05. (**B**) Huh7 cells were transfected with control siRNA (NC) or TNF-α siRNA for 48 h followed by the transfection of siRNA again for another 6 h. Cells were then infected with HCV for 24 h and then treated with IFNα for another 24 h before lysis for immunoblot analysis. (**C**) Huh7 or KO-I613 cells were infected with HCV (MOI = 0.25). The cells were lysed at indicated points followed by immunoblot analysis.

HCV could activate TLR7 and TLR8 early in infection to induce the expression of TNF-α (9, 25). This induction of TNF-α is important to support IFN signaling as it prevents the depletion of IFN-α and -β receptor subunit 2 (IFNAR2) by HCV (9). For that reason, we also investigated the possible effect of PKD1 on the induction of TNF-α in HCV-infected cells. As shown in Fig. 6A, HCV increased the expression level of TNF-α approximately 10-fold in Huh7 cells, which was consistent with the previous reports. Interestingly, comparing KO-I613 cells with Huh7 cells, the knockout of PKD1 increased the expression level of TNF-α approximately 5-fold, which was further increased to more than 30-fold when cells were infected by HCV. These results indicated that PKD1 suppressed the expression of TNF-α, and its knockout and HCV infection generated a synergistic stimulatory effect on the expression of TNF-α.

STAT1 mediates IFN signaling and is phosphorylated and activated by the binding of type I IFNs to the IFN receptor (IFNAR) (26). As shown in Fig. 6B, there was very little phosphorylation of STAT1 in Huh7 cells and its phosphorylation was not affected by HCV at 24 h post-infection or by the silencing of TNF-α. In contrast, the treatment of Huh7 cells with IFN-α led to the phosphorylation of STAT1, which was further enhanced by HCV infection. The treatment of IFN-α also led to the loss of the HCV core protein (Fig. 6B). However, if the expression of TNF-α was silenced by siRNA, the effect of IFN-α on STAT1 was diminished, resulting in a partial restoration of the HCV core protein level (Fig. 6B). These results indicated that IFN-α signaling and its effect on HCV were partially dependent on TNF-α.

To further determine the effect of PKD1 on TNF-α and IFN signaling, we examined the effect of PKD1 on TNFR1 and the phosphorylation of STAT1. As shown in Fig. 6C, HCV reduced the protein level of TNFR1 to an undetectable level after 48 h of infection (Fig. 6C). In agreement with the results shown in Fig. 6B, the phosphorylation of STAT1 was not detected during the first 48 h of HCV infection although a strong induction of its phosphorylation was detected at 72 h post-infection, which was correlated with the reduction of the HCV core protein level. The marked induction of STAT1 phosphorylation at 72 h might be associated with the substantial reduction in PKD1 phosphorylation observed at this timepoint (Fig. 1A). PKD1 knockout increased the expression of TNFR1 in mock-infected cells, and this increase was associated with the induction of STAT1 phosphorylation (Fig. 6C), again indicating a role of PKD1 in suppressing IFN signaling. HCV infection led to the gradual loss of TNFR1 from KO-I613 cells in a time-dependent manner although a strong induction of STAT1 was also observed at 72 h post-infection, presumably due to the induction of IFNs by HCV (Fig. 6B). The activation of STAT1 at this timepoint was also associated with the further reduction of the HCV core protein level. PKD1 knockout did not affect the IFNAR2 level in the absence of HCV infection. However, it increased the IFNAR2 level at 24 and 48 h post-infection, likely due to enhanced TNF-α/TNFR1 signaling, which prevented the depletion of IFNAR2 by HCV (Fig. 6C). Taken together, the results shown in Fig. 6 indicated that PKD1 suppressed the expression of TNF-α and its receptor and, as such, reduced the expression of IFNAR2 and the sensitivity of HCV-infected cells to IFNs.

### PKD1 enhances the inflammasome response and pyroptosis to suppress IFN response

**Fig 7:**
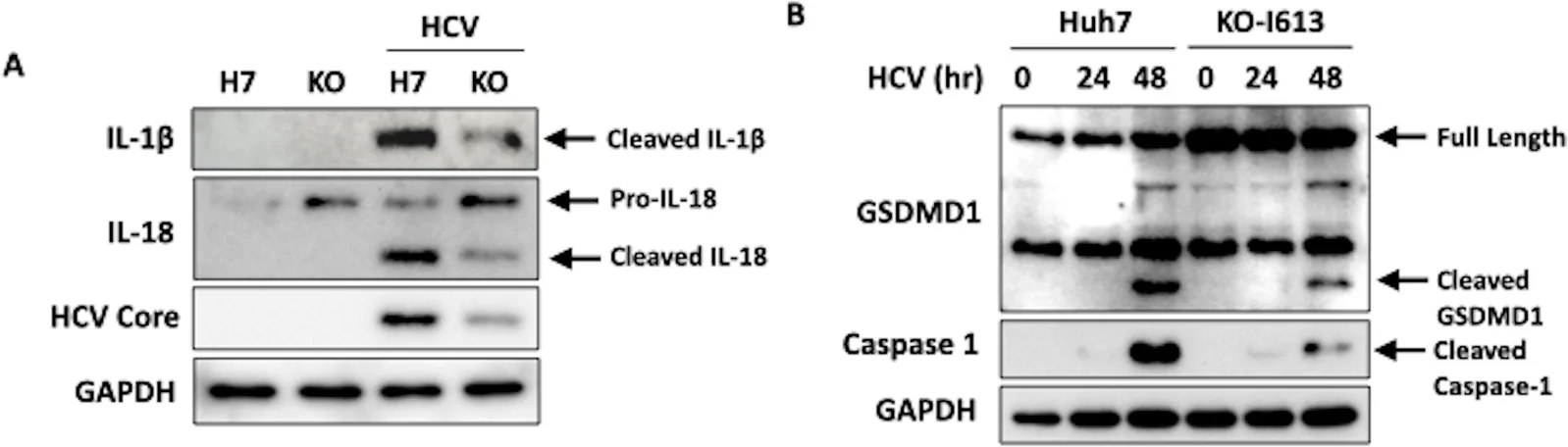
PKD1 promotes the inflammasomal response induced by HCV. (**A and B**) Huh7 or KO-I613 cells were infected with HCV (MOI = 1). After 4 h, the inoculum was removed and replaced with fresh maintenance media. The cells were further incubated for 24 h (**A**) or 24 or 48 h (**B**) and then lysed for immunoblot analysis.

Inflammasome is a multiprotein complex, which, upon activation, triggers the autocleavage of procaspase-1 to generate caspase-1. Caspase-1 will then cleave pro-IL-1β and pro-IL-18 to generate IL-1β and IL-18, respectively, for their subsequent release. IL-1β and IL-18 are pro-inflammatory cytokines. HCV had previously been shown to activate inflammasomes in macrophages (27, 28) and hepatocytes (29–31). For that reason, we also examined the possible role of PKD1 in HCV-induced inflammasomal response. As shown in Fig. 7A, in agreement with the previous reports, HCV infection of Huh7 cells led to the production of mature IL-1β and IL-18, which are hallmarks of the activation of inflammasomes. This induction of mature IL-1β and IL-18 by HCV was attenuated in PKD1-knockout cells, indicating an important role of PKD1 in the induction of inflammasomes. This possibility was further supported by our analysis of caspase-1 and gasdermin D1 (GSDMD1). Caspase-1, once it is activated, can cleave GSDMD1, which will then form pores on plasma membranes for the release of mature IL-1β and IL-18 and ultimately lead to cell death in a process known as pyroptosis (32). As shown in Fig. 7B, HCV could induce the cleavage of GSDMD1 in Huh7 cells at 48 h post-infection. This cleavage of GSDMD1 coincided with the activation of caspase-1. However, the ability of HCV to activate caspase-1 and induce the cleavage of GSDMD1 was reduced in KO-I613 cells. These results together indicated that, in addition to suppressing the expression of TNF-α and attenuating IFN signaling, PKD1 also mediated the inflammasomal response and pyroptosis.

## DISCUSSION

**Fig 8:**
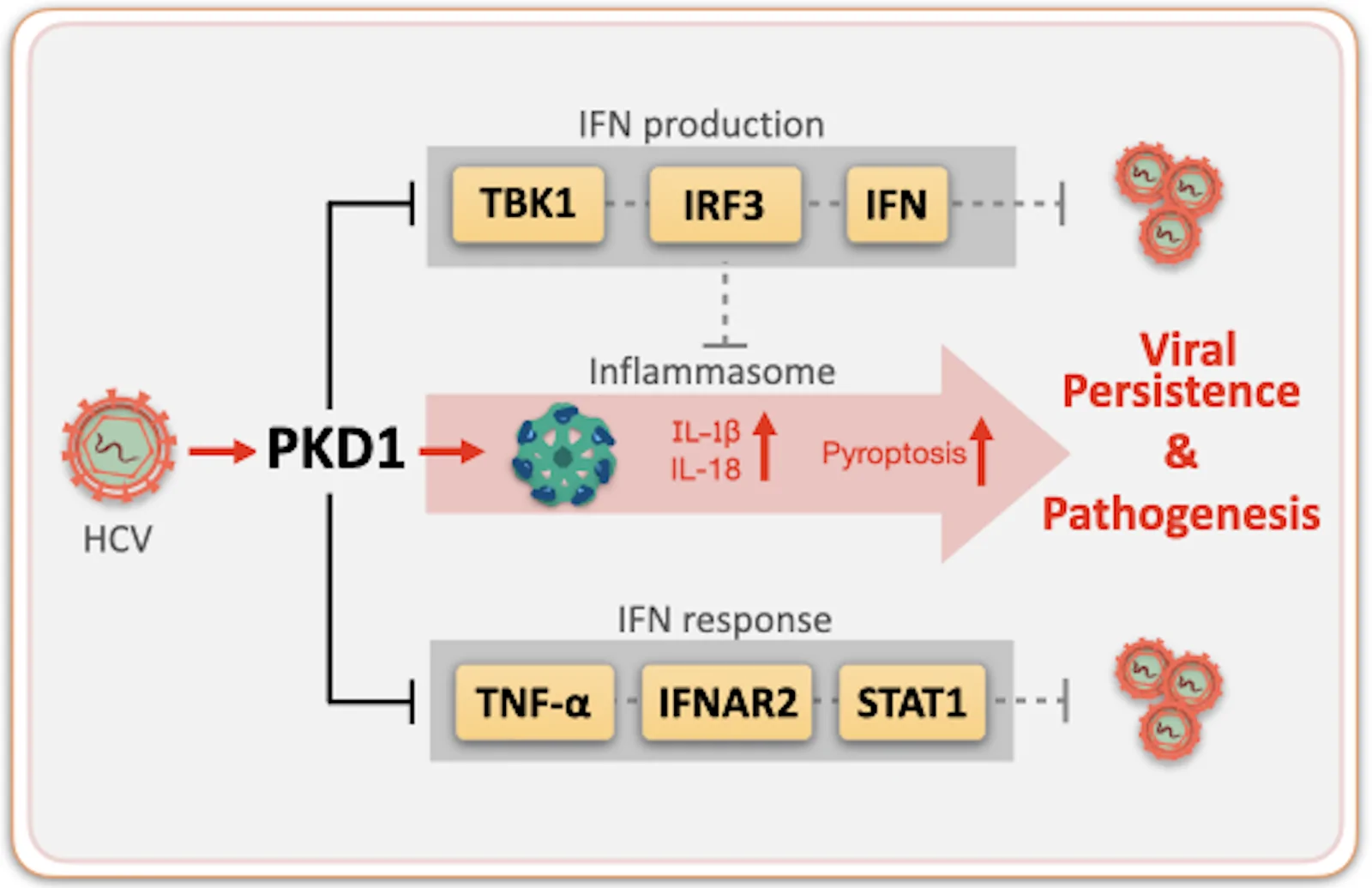
HCV-activated PKD1 attenuates IFN and TNF-α signaling pathways and induces inflammasomes to promote HCV persistence and pathogenesis. HCV-activated PKD1 promotes HCV replication and pathogenesis through multiple mechanisms. It suppresses type I IFN production by inhibiting TBK1-IRF3 signaling. It also inhibits TNF-α production, which supports IFNAR2 expression to sensitize cells to IFN-α. These effects of HCV on PKD1 promote HCV replication. HCV-activated PKD1 can also stimulate inflammasomal response, ultimately leading to pyroptotic cell death and liver tissue damage.

PKD1 is involved in multiple cellular pathways including proliferation, lipid metabolism, and Golgi-associated secretion processes (14). In the present study, we showed that HCV could activate PKD1 via PKC in the early stage of its infection (Fig. 1) and induce its cleavage via caspase-3 in the late stage of infection (Fig. 2). PKD1 did not affect HCV entry into its host cells (Fig. 4A) or the HCV IRES activity (Fig. 4D). However, it enhanced HCV replication both in cells infected by HCV (Fig. 3) and in cells harboring an HCV subgenomic RNA replicon (Fig. 4B and C). Our further studies indicated that, by activating PKD1, HCV restricted the activation of TBK1 and its downstream effector IRF3 to suppress the production of IFNs and ISGs (Fig. 5). The relationship between HCV-activated PKD1 and the TBK1-IRF3-IFN pathway is summarily illustrated in Fig. 8. How PKD1 negatively regulated TBK1 and IRF3 is unclear, and it will be interesting to determine whether this is due to its physical interaction with TBK1 and/or its kinase activities. Amako et al. previously showed that PKD1 suppressed the secretion of progeny HCV particles from HCV-infected cells by inhibiting ceramide transfer protein (CERT) and oxysterol binding protein (OSBP) that otherwise promoted HCV secretion (18). Our results now showed that PKD1 also played an important role in the suppression of IFN signaling in the early phase of HCV infection to promote HCV replication. Based on their results and ours, it is conceivable that HCV activates PKD1 to inhibit IFN immune response to promote its replication in the early phase of infection and induced the cleavage of PKD1 to promote the release of its progeny viral particles from infected cells in the late phase of infection.

Our finding that PKD1 could negatively regulate TNF-α expression (Fig. 6A) indicated that, in addition to suppressing IFN signaling, HCV could also activate PKD1 to inhibit the expression of this cytokine. This finding is important, as TNF-α supports IFN signaling, and the inhibition of its expression can attenuate the IFN response of HCV-infected cells (Fig. 6B). In addition, TNF-α can also activate caspase-8 and subsequently caspase-3 to trigger extrinsic apoptosis (Fig. 1A) (33). The activation of PKD1 to reduce the expression of TNF-α can suppress this process to prolong cell survival and HCV replication. There is also an interesting interplay between PKD1 and TNF-α since the former can suppress the expression of the latter (Fig. 6A), and the latter can cleave the former via the activation of caspase-3 (Fig. 2). Thus, the tilt of the balance in the battle between PKD1 and TNF-α can determine the fate of HCV-infected cells.

Previous studies indicated that TNF-α induced by HCV was necessary for type I IFN signaling in HCV-infected cells, as the silencing of either TNF-α or its receptor TNFR1 abrogated the expression of IFNAR2, a subunit of the type I IFN receptor, and desensitized HCV-infected cells to type I IFNs (9, 34). Thus, in addition to directly suppressing IFN signaling, HCV may also indirectly suppress IFN signaling by attenuating the expression of TNF-α via the activation of PKD1. This possibility is supported by two lines of evidence: first, the silencing of TNF-α inhibited the activation of STAT1 by IFN-α in HCV-infected cells (Fig. 6B), and second, the loss of PKD1, which enhanced the expression of TNF-α (Fig. 6A), also induced the activation of STAT1 in HCV-infected KO-I613 cells (Fig. 6C). These findings are consistent with the model that HCV activates PKD1 to attenuate the expression of TNF-α and desensitize HCV-infected cells to type I IFNs (see Fig. 8).

HCV had previously been shown to activate inflammasomes in hepatocytes (29–31). We were able to confirm this previous finding and further demonstrate that PKD1 mediated this inflammasomal response to promote the production of mature IL-1β and IL-18 as well as the cleavage of GSDMD for the induction of pyroptosis (Fig. 7A and B). Note that, in contrast to our finding and those reported by others, Chen et al. reported that HCV could not induce inflammasomes in Huh7 cells (28). The reason for this discrepancy is unclear and might be due to the use of different HCV strains, as Chen et al. used the parental HCV JFH1 strain that did not replicate robustly in cell cultures. Type I IFNs via the activation of STAT1 can repress the activities of NLRP1- and NLRP3-inflammasomes to inhibit the production of mature IL-1β (35). In addition, the activation of STAT-1 can also induce the expression of IL-10, which then activates STAT-3 via an autocrine mechanism to suppress the expression of pro-IL-1α and pro-IL-1β (35). Based on these previous findings, it is likely that the loss of PKD1 suppressed the inflammasomal response and the ensuing pyroptosis via the induction of type I IFNs and the autocrine activation of STAT1. It is conceivable that PKD1 by promoting inflammatory response in HCV-infected cells also contributes to the development of HCV-related pathogenesis (Fig. 8). It should be noted that the induction of pyroptosis had also been suggested to facilitate the release of intracellular HCV particles (30).

Evasion of the host innate immune response by HCV is essential to establish chronic infection in patients. The intricate balance between the inflammatory response and the interferon response can have profound impacts on HCV persistence and pathogenesis. Our findings that HCV could regulate PKD1 to suppress antiviral responses and promote inflammasomal responses provide an explanation as to why most patients infected by HCV fail to clear this viral infection and ultimately develop severe liver diseases.

## MATERIALS AND METHODS

### Cell cultures and HCV preparation

Huh7 cells were maintained in Dulbecco’s modified Eagle medium (DMEM) supplemented with 10% fetal bovine serum (FBS) and Penicillin/Streptomycin/Amphotericin B Solution. Huh7.5 cells were maintained in DMEM supplemented with 10% FBS, 1% nonessential amino acids, and Penicillin/Streptomycin/Amphotericin B Solution. Primary human hepatocytes (PHHs) were obtained from the Cell Culture Core Facility of the USC Research Center for Liver Diseases. They were maintained in DMEM medium supplemented with 10% FBS. The cell culture-adapted JFH1 (HCV genotype 2a) variant, which produced a high level of infectious virus particles (19), was propagated in Huh7.5 cells and used in our infection studies. Sg-PC2 cells were derived from Huh7 cells and contained an HCV subgenomic RNA replicon that contained both the neomycin-resistant gene and the HCV NS3-NS5B sequence. The establishment of this cell line had been described (23). This cell line was maintained in DMEM containing 10% FBS, Penicillin/Streptomycin/Amphotericin B, and G418 (400 μg/mL).

### Immunoblot analysis and antibodies

Cells were lysed in M-PER Mammalian Protein Extraction Reagent (Thermo Fisher Scientific) containing the protease inhibitor cocktail (Halt Protease and Phosphatase Inhibitor Cocktail, Thermo Fisher Scientific) for 10 min on ice followed by a brief sonication. Cell lysates were cleared by centrifugation at 14,000 × *g* for 2 min. The supernatant was collected, boiled in Laemmli buffer for 5 min, and used for immunoblot analysis or stored at −80°C for future use. The protein lysates of primary human hepatocytes analyzed here were generated as part of a previously published study (9). The rabbit anti-HCV core antibody was prepared in our laboratory (36). Antibodies to phospho-PKD/PKCμ (Ser744/748), PKD/PKCμ, phospho-TBK1 (Ser172), TBK1, phospho-IRF3 (Ser396), IRF3, Caspase-8, Caspase-3, PARP, GAPDH, phospho-STAT1(Y701), and STAT1 were from cell signaling. The actin antibody was from Sigma. The mouse monoclonal anti-HA antibody was from Santa Cruz. Horseradish peroxidase-conjugated goat anti-rabbit and rabbit anti-mouse secondary antibodies were also purchased from Abcam.

### Immunofluorescent microscopy

Huh7 cells or KO-I613 cells were seeded on the cover slip that was placed in the well of a 6-well plate. The cells were fixed with either paraformaldehyde (4%) for 20 min or cold acetone (−20°C) for 2 min. The fixed cells were blocked and permeabilized with blocking/permeabilizing buffer (4% bovine serum albumin [BSA] and 0.3% Triton X-100 in PBS) for 1 h at room temperature. The cells were then incubated with the primary antibody overnight at 4°C. The next day, the cells were washed with PBS containing 1% BSA, 0.02% saponin, and 0.05% sodium azide. The cells were then incubated with secondary antibody for 1 h at 37°C. After washing two times with the washing buffer and two times with PBS, the coverslips were mounted on slides. The images were taken with Keyence fluorescence microscope BZ9000.

### Generation of CRISPR/Cas9-knockout cell line

To generate PKD1 knockout cells, a guide RNA sequence pair targeting exon 2 of the human PKD1 gene (5′-CACCGTTCCGTAGAAACCACATTC-3′ and 5′-AAACTGAATGTGGTTTCTACGGAAC-3′) was cloned into the plasmid vector PX459 (Addgene, #48138) that expressed SpCas9 and the puromycin resistance gene. Briefly, the pair of guide DNA (gDNA) was annealed and phosphorylated at the 5′-end using T4 polynucleotide kinase. Then, the gDNA was cloned into the PX459 plasmid vector. The correct insertion of the gDNA into PX459 was verified by nucleotide sequencing. Huh7 cells were then transfected with the PX459/gDNA plasmid. At 48 h post-transfection, Huh7 cells that contained the PX459/gDNA plasmid were selected with puromycin for 72 h. Single cell clones were then selected by limiting dilution in a 96-well dish. The genomic DNA of selected colonies was sequenced to check for the disruption of the gene by CRISPR/Cas9. The depletion of PKD1 protein was confirmed by immunoblot. Additional information and the results of the selection process are shown in the supplemental information in Fig. S1.

### Gene silencing

The siRNA targeting PKD1 was from Sigma. The transfection of siRNA into Huh7 cells was performed using Lipofectamine 2000 (Invitrogen). Briefly, Huh7 cells seeded in 100 mm dishes were transfected with 600 pmole siRNA for 6 h. The transfected cells were further incubated in fresh media for 48 h prior to a second-time transfection with 600 pmole siRNA. After 6 h, the siRNA/lipofectamine complex was replaced with HCV-containing media. The cells were then lysed 24 h later.

### Semi-quantitative PCR and quantitative reverse transcription-PCR

Total cellular RNA was isolated using TRIzol (Invitrogen) and reverse-transcribed to cDNA using SuperScript II First-Strand Synthesis System for RT-PCR (Invitrogen). The cDNA was then subjected to PCR amplification with the primer sets listed in Table S1. For the detection of gene expressions using real-time PCR, 100 ng total RNA was analyzed using the *Power* SYBR Green RNA-to-CT 1-Step Kit (Applied Biosystems, Foster City, CA). The primers used are shown in Table S1. For the quantification of HCV RNA, total cellular RNA was subjected to quantitative reverse transcription-PCR (qRT-PCR) using the TaqMan Fast Virus 1-Step Master Mix (ThermoFisher Scientific) following the manufacturer’s instructions. HCV JFH1 primers 5′-TCTGCGGAACCGGTGAGTA-3′ (forward) and 5′-TCAGGCAGTACCACAAGGC-3′ (reverse) and the probe 5′-CACTCTATGCCCGGCCATTTGG-3′ were used for the qRT-PCR. The control GAPDH primer set with the probe was purchased from Applied Biosystems.

### Bicistronic HCV RNA analysis

The plasmid pRL-HL, which expresses a bicistronic RNA coding for renilla luciferase at the 5′-end and firefly luciferase at the 3′-end, had been reported before (37). The translation of the renilla luciferase in this construct was cap-dependent, whereas that of the firefly luciferase was mediated by the HCV IRES. Huh7 cells were transfected with either negative control (NC) siRNA or PKD1 siRNA. After 48 h, the cells were transfected with the corresponding siRNA together with the pRL-HL plasmid and incubated for an additional 48 h before lysis. Cell lysates were analyzed using the Dual-Luciferase Reporter Assay System (Promega) according to the manufacturer’s instructions.

### Statistical analysis

All data were analyzed using an independent-samples, two-tailed *t*-test. Results are presented as the mean **±** standard deviation. Differences with *P* < 0.05 were considered statistically significant and denoted by an asterisk (*), whereas differences with *P* ≥ 0.05 were considered not statistically significant and denoted as ns. All immunoblot studies were repeated at least three times to ensure the reproducibility of the results.

## Data Availability

All original data supporting the findings of this study will be provided upon reasonable request.

## SUPPLEMENTAL MATERIAL

Supplemental material jvi.00693-26-s0001.pdf Table S1; Fig. S1 and S2.

ASM does not own the copyrights to Supplemental Material that may be linked to, or accessed through, an article. The authors have granted ASM a non-exclusive, world-wide license to publish the Supplemental Material files. Please contact the corresponding author directly for reuse.
