# Supplementary material for: Protein kinase D1 regulates innate immune signaling pathways to promote hepatitis C virus replication
Source: J Virol. 2026 Sep 9;100(10):e00693-26. doi: 10.1128/jvi.00693-26 (PMC13712750; doi:10.1128/jvi.00693-26)

Supplementary Information

Table 1. Primers used in PCR reactions.

Primers for PCR			
Gene	Forward	Reverse	
GAPDH	GGAGCGAGATGGCTCCAAAAT	GGCTGTTGTCATACTTCTCAT	
IFNαA5	GCTCAACTGCAAGTCAATCTG	CTTGAGCCTTCTGGAACTGG	
IFNβ1	TGACCAACAAGTGTCTCCTC	GAATCCAAGCAAGTTGTAGCT	
MxA	GCAATGATCCCAAAGTAGACC	GTTTCCGAAGTGGACATCGCA	
OASL	CTGATGCAGGAACTGTATAGC	CTGATGCAGGAACTGTATAGC	
ISG15	GCAGATCACCCAGAAGATCG	TTCGTCGCATTTGTCCACCA	
TNF-α	ATCAATCGGCCCGACTATCTC	GCAATGATCCCAAAGTAGACC	
IL-1β	AGCTACGAATCTCCGACCAC	CGTTATCCCATGTGTCGAAGAA	

Supplemental Figure Legends

Figure S1. Efficiency of PKCδ silencing.

Huh7 cells transfected with either control siRNA or PKCδ siRNA, as shown in Figure 1D, were lysed for total RNA isolation. The RNA was used to quantify PKCδ and GAPDH mRNA levels by Sybr Green reverse transcription PCR (RT-PCR). PKCδ mRNA expression was normalized to that of GAPDH.

Figure S2. Validation of PKD1 knockout in KO-I613 cells.

Single clones of Huh7 cells transfected with PX459/gDNA plasmid were isolated by limiting dilution. (A) Comparison between the parental Huh7 cells and two selected cell clones. Immunoblot analysis of PKD1 revealed a successful depletion of PKD1 in clone 6 and a partial depletion of PKD1 in clone 7. When these cells were infected by HCV, a reduction of HCV core protein level was observed in both clones 6 and 7 when they were compared against the parental Huh7 cells. (B) Clone 6 was further analyzed for verification of the disruption of the PKD1 gene. The chromosomal DNA sequence of clone 6 encompassing the target sequence of gRNA was amplified by PCR and then digested with the restriction enzyme Psr I. The PCR amplicon from the parental Huh7 genomic DNA was cut by Psr I whereas the PCR amplicon from clone 6 was not, confirming that the area surrounding Psr1 restriction site was disrupted by CRISPR/Cas-9. Clone 6 was subjected to further purification by limiting dilutions for the isolation of its derivative KO-I613. (C) The sequencing result of KO-I613 confirmed that it contained multiple mutations in the PKD1 sequence that was targeted. These mutations abolished the expression of PKD1. The locations of the gRNA and the PsrI site are indicated.

Figure S1. Efficiency of PKCδ silencing.

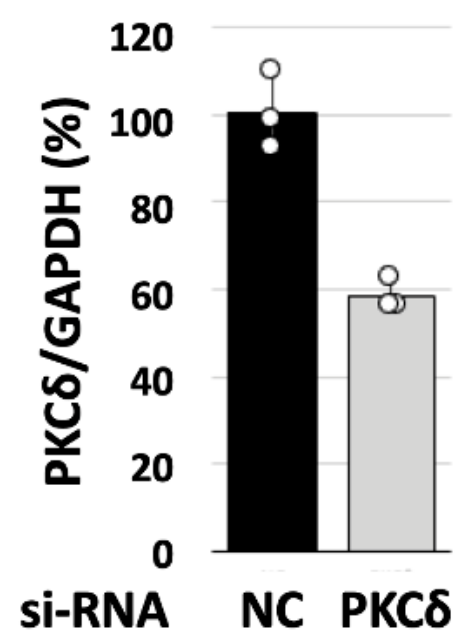

Figure S2. Validation of PKD1 knockout cells.

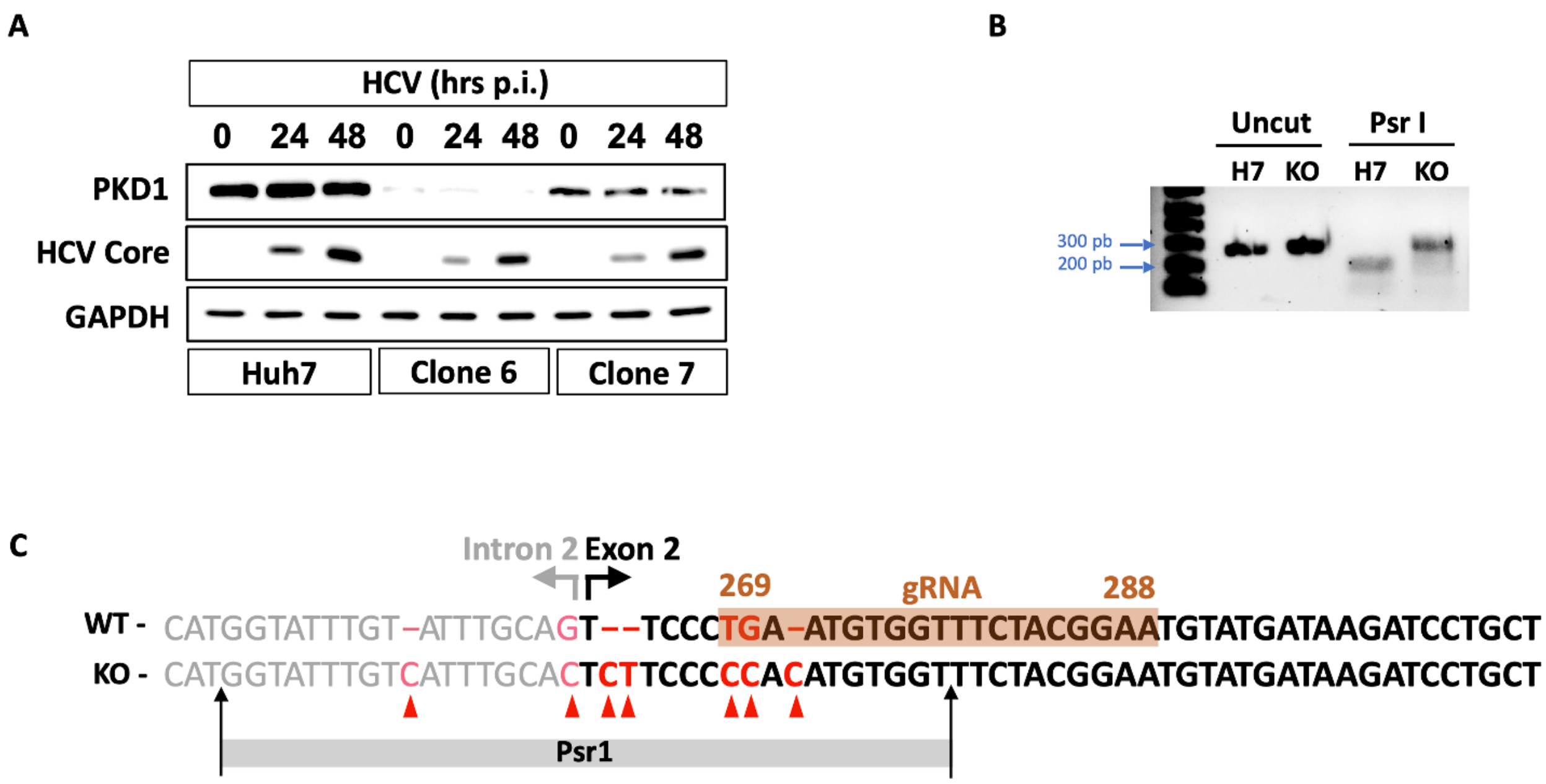

Supplement: Supplementary file 1 [file jvi.00693-26-s0001.pdf]
